# Cell-specific copper dyshomeostasis mechanism in Alzheimer’s disease

**DOI:** 10.1186/s40035-025-00504-6

**Published:** 2025-08-22

**Authors:** Michael Okafor, Peter Faller, Nicolas Vitale

**Affiliations:** 1 Laboratory of Biometals and Biological Chemistry, Institut de Chimie (UMR 7177), Université de Strasbourg-CNRS, 4 Rue Blaise Pascal, 67000 Strasbourg, France; 2 Centre National de la Recherche Scientifique, Université de Strasbourg, Institut des Neurosciences Cellulaires et Intégratives, 67000 Strasbourg, France

**Keywords:** Alzheimer's disease, Amyloid, Copper, Neurodegeneration, Oxidative stress

## Abstract

Alzheimer's disease (AD) is a complex neurodegenerative disorder characterized by progressive decline of cognitive functions, yet its underlying aetiology remains elusive. While amyloid-β (Aβ) and tau pathologies have been extensively studied, emerging evidence suggests that metal and especially copper dyshomeostasis may also play a crucial role in the pathogenesis of AD. This review explores the intricate relationship between copper and AD, shedding light on the multifaceted mechanisms through which copper dysregulation contributes to neurodegeneration. We delve into the impact of copper ions on Aβ aggregation, tau phosphorylation, and oxidative stress, providing a comprehensive overview of the molecular pathways involved. Furthermore, we discuss the interplay between different brain cell types and the impact Cu dysregulation may have on them. The therapeutic implications of targeting copper dysregulation for AD treatment are also addressed, emphasizing the potential of copper-modulating agents in ameliorating cognitive decline. In summary, this review discusses copper dyshomeostasis as a central player in the intricate tapestry of AD pathology, offering new insights and avenues for therapeutic interventions.

## Introduction

Alzheimer’s disease (AD) is the leading cause of dementia as well as the leading neurodegenerative disease at present. Although we have gained some insights into some of the underlying mechanisms of the disease through intense research effort, there is still no cure after over a hundred years of research. The first known description of AD was published by the German psychiatrist, Alois Alzheimer in 1907, with a more comprehensive description a few years later, independently by Solomon Carter Fuller and Alois Alzheimer [1–3]. They commented on the presence of aggregates, inside and outside neurons, known today as neurofibrillary tangles (NFTs) and amyloid plaques, respectively [2]. These represent the major hallmarks of AD. Amyloid plaques can vary in structure and composition, with the main two being diffuse and neuritic plaques composed primarily of Aβ. Diffuse plaques tend to be non-fibrillary, loose and can also be present in people without dementia [4]. Neuritic plaques on the other hand, have a dense core, constituted of Aβ surrounded by dystrophic neurites. In addition to the plaques in the brain, up to 90% of AD patients also have Aβ deposited in cortical blood vessels (cerebral amyloid angiopathy) [5, 6]. This leads to degeneration of vascular smooth muscle and weakening of the blood-brain barrier (BBB), leading to microinfarctions and haemorrhages [7].

Despite the countless evidence of the accumulation of amyloid plaques in AD, tau load seems to show a better correlation with disease progression. Thus, the progression of AD was categorised into six Braak stages according to the spread of NFTs: stage I–II with NFTs in the entorhinal cortex and hippocampus, III–IV in the limbic lobe, amygdala and fusiform gyrus, V in the associated areas of the neo cortex, and VI in the primary sensory and motor areas [8, 9]. Physiologically, tau proteins are found at the axons of neurons where they stabilise microtubules to permit transport of cargoes. However, recent work shows that, unlike the established dogma, in adult human brain cortical neurons tau is abundant within the somatodendritic compartments compared to axons [10]. Tau possesses many sites for phosphorylation, and tau phosphorylation is increased in the CSF of AD patients [11]. In AD, hyperphosphorylated tau is present as paired helical filaments (PHFs) and aggregates in neurons forming NFTs [12].

## Amyloid precursor protein (APP), a major player?

### Different forms of AD

AD can be classified under two general families, familial (fAD) and sporadic (sAD). fAD, the familial form, also known as early-onset, is the hereditary form as it can be transferred from parents to offspring. fAD accounts for an estimated 1% of AD patients and presentation of symptoms usually occurs before the age of 65 [13, 14]. All forms are autosomal dominant with a high degree of penetrance. Mutations in genes involved in APP metabolism, such as *APP*, *PSEN1* (*PS1*) and *PSEN2* (*PS2*) have been shown to induce fAD.

APP is a transmembrane protein with functions still not well defined, although it is thought to be a surface receptor and is involved in synaptic plasticity, neurite outgrowth as well as adhesion amongst others [15]. Interestingly, *APP* does not seem to be a vital gene in neurodevelopment, as *APP* knockout mice do not display any physical and cognitive defects in comparison to wild-type mice [24]. However, after 14 weeks of age there is abnormal reactive gliosis in the hippocampus and neocortex of these mice, accompanied by decreased locomotor activity [16]. It would take the deletion of the APP family genes (*APP*, *APLP1*, *APLP2*) all together for lethality to occur. This hints at some kind of compensatory action between these genes for different functions [17, 18].

**Fig. 1:**
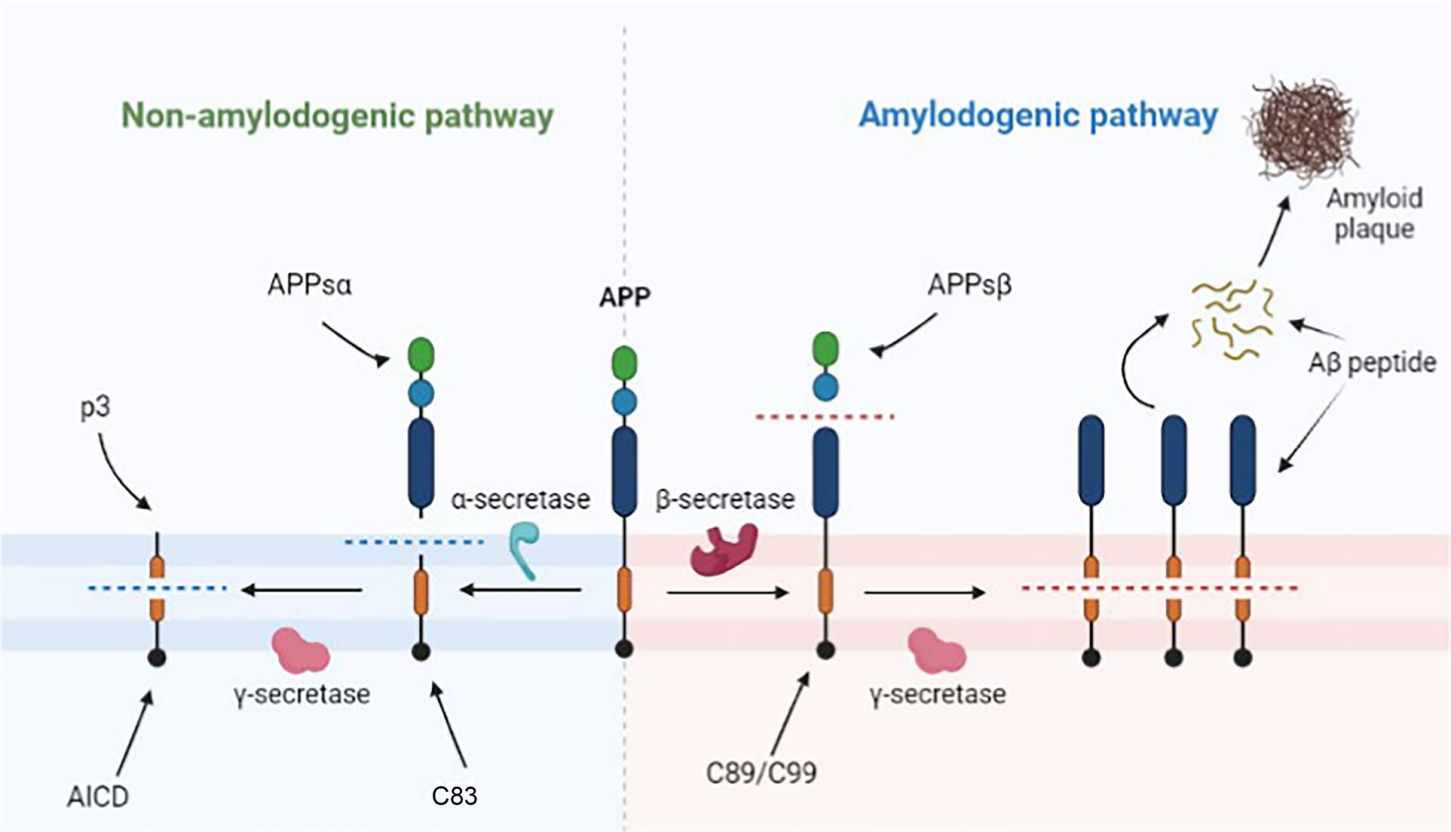
Representation of the alpha and beta metabolic pathways of APP. Dashed lines represent cleavage by secretases

In the secretory pathway, APP is naturally subject to two distinct metabolic processing pathways, namely the alpha and the beta pathways, with the former being the dominant and primary pathway under non-pathological conditions [19]. In the beta pathway, APP is first cleaved by β-secretases (optimal activity in low pH [20]) mostly at the Asp1 residue or less frequently at Glu11, resulting in two distinct fragments (Fig. 1), APPsβ and C89/C99. The latter is then cleaved in the intra-membrane region by γ-secretases to form the intracellular domain of the amyloid precursor protein (AICD) and the Aβ_11-40/42_ or Aβ_1-40/42_ peptide [21]. BACE1, the major β-secretase in the beta pathway, is mainly found in the endosomes and the trans-Golgi network (TGN) where it preferentially generates the Aβ peptides. However, it has been shown that BACE1 can also cleave APP in the endoplasmic reticulum (ER) [21]. On the other hand, the alpha pathway first involves α-secretases cleaving the intramembrane segment of Aβ to form APPsα (neurotrophic and neuroprotective properties) and C83, mainly at the plasma membrane or TGN [21, 22]. C83 is then cut by γ-secretases to form AICD and p3 fragments [23].

The Aβ_40_/Aβ_42_ ratio from the beta pathway can be altered by mutations in genes involved in APP processing. Mutations outside the Aβ regions on APP lead to an increase in β-secretase activity, whereas mutations in the Aβ regions usually lead to local oligomer formation and changes in the interactions with proteins such as APOE and LRP1, and affect their clearance [24]. It has also been shown that posttranslational modifications such as phosphorylation can change the proportions of Aβ secreted. Phosphorylation of APP at Ser655 permits retrograde transport of APP from endosomes to TGN and disfavours lysosome targeting [25]. The phosphorylation at Ser655 can be carried out by GSK, protein kinase C (PKC), CaMKII (calcium–calmodulin (CaM)-dependent protein kinase II) or APP kinase 1 and dephosphorylation by PP1 and PP2 [26–28]. Conversely, phosphorylation at Thr668 occurs on immature APP in the ER and permits ER retention [29].

Nearly 90% of fAD are linked to mutations in *PS1* and *PS2*, leading to a defect in APP processing [30, 31]. However, they are also involved in the cleavage of Notch-1, N- and E-cadherin, LRP, Syndecan, Delta, Jagged, CD44, ErbB4, and Nectin1a [30]. PS1/PS2 are cleaved to form an N- and a C-terminal fragment and are the catalytic core of the γ-secretase complex along with Nicastrin, Aph-1 and Pen-2. Counterintuitively, the mutations in *PS*1/*PS*2 as seen in fAD lead to a decrease in the γ-secretase activity [32, 33]. Sun et al. tested in vitro 138 mutations in the human *PS1* gene known to lead to AD and found that 90% of these mutations led to reduced production of Aβ_40_ and Aβ_42_ but with an overall increase in the Aβ_42_/Aβ_40_ ratio [34].

In sAD, the greatest risk factor for the development of AD is age. The chance of developing AD doubles every 5 years after 65 and reaches up to 50% after 85 years of age [35]. However, there is also an array of both environmental and genetic factors that lead to the development of AD. The environmental factors include socioeconomic status, smoking, diabetes, hypertension, high cholesterol levels, etc. [36]. Equally, there is an increasing number of gene polymorphs that are strongly associated with sAD, including *APOE*-ε4, *TREM2* and *PLD3* variants amongst others [37]. The highest genetic risk factor in the development of AD is the *APOE*-ε4 variant [38, 39]. The human ApoE exists in 3 isoforms, ApoE2, ApoE3 and ApoE4, with ApoE3 being the most common [40]. In mice, ApoE is expressed in glial cells under normal conditions, but during stress it is also produced by neurons [41]. However, in humans, transcription of *APOE* has also been detected in neurons of the cortex and hippocampus [42]. ApoE is involved in the uptake and distribution of lipids and has been shown to bind to Aβ and influence its removal from the brain [43]. ApoE2 has the best lipid efflux activity in astrocytes and neurons, followed by ApoE3, then ApoE4 [44]. Accordingly, impaired interaction of low-density lipoprotein receptor (LDLR) and lipidated ApoE2 prevents LDLR recycling defects observed with lipidated ApoE3/E4, and decreases the uptake of cholesteryl esters, lipids linked to neurodegeneration [45]. In AD, *APOE*-ε4 carriers generally have more plaques and tangles than carriers of other isoforms, with > 65% of AD patients having an *APOE*-ε4 allele [40, 46]. Presence of both alleles of *APOE*-ε4 increases the chances of AD to 91%, compared to 20% in patients with one copy of *APOE*-ε2 or *APOE*-ε3 [47].

Recently, a possible link between sAD and fAD was suggested. Hou et al. showed that ApoE has the capacity to interact with and inhibit γ-secretase activity through its C-terminal region, leading to a significant decrease of Aβ_40_ and Aβ_42_ production but not APPsβ (β-secretase activity) [48]. The effectiveness of this inhibition inversely correlates with the risk nature of *APOE,* with ApoE2 having the most efficient inhibitory activity followed by ApoE3 and ApoE4, which has no inhibitory activity [48]. Of note, *APOE*-ε2 is known to be protective against the development of AD [40].

Another important genetic risk factor is the presence of certain variants in *TREM2* gene. TREM2 is a transmembrane surface protein found on microglia that has also been shown to be important in the initial seeding phase of amyloid plaques. Previous studies have shown that TREM2 is a receptor for Aβ oligomers [49, 50]. Different variants of *TREM2*, p.R47H, R62H, H157Y, and D87N, are known to increase the risk of AD by 2–4 folds [51–54]. Furthermore, the absence or loss of function of TREM2 causes a decrease in plaque phagocytosis [50, 55]. TREM2 acts to compact amyloid plaques, reducing the perimeter covered by the plaques and therefore likely lowering plaque-induced toxicity. Accordingly, in the absence of TREM2, amyloid plaques are more diffuse with a loose structure [55, 56]. Interestingly *Trem2*-deficient mice display similar phenotypes compared to *Apoe*-deficient mice, bringing to light the APOE-TREM2 axis [57]. All the factors mentioned above provide support for the “amyloid hypothesis”.

### Amyloid hypothesis

The amyloid hypothesis was coined by Hardy and Higgins, 1992, who proposed that AD is caused by the presence of amyloid plaques in the brain, leading to the emergence of NFTs, cell loss, vascular damage, and dementia [58]. This theory came into fruition thanks to the discovery by George Glenner and Caine Wong, where they identified Aβ as the central molecule of amyloid plaques found in AD and trisomy 21 (Down’s syndrome) [59, 60].

The backbone of the amyloid hypothesis lies on the observation that in AD, there is abnormal APP metabolism which results in increased Aβ peptide in the brain and the blood [61]. This alteration in APP metabolism could arise from any or a combination of the following: increased expression of APP, resulting in increased metabolites (both alpha and beta pathways); or an abnormal increase in the beta processing pathway, resulting in increased Aβ production; or simply from a defect in Aβ clearance. Previous studies have shown defects in the activity and levels of neprilysin and insulin degrading enzyme (IDE) in AD, proteases known to efficiently cleave Aβ [62–64]. Miners et al. (2009) reported an increased activity of IDE and neprilysin in the brains of AD patients [65]. This signifies that there is an upregulation in the clearance of Aβ in AD, but it might be inefficient due to the presence of Aβ in different conformations (aggregates for example). Another key factor is the clearance of Aβ by transporters from the brain. Export of Aβ from the brain is primarily attributed to low-density lipoprotein (LDL) receptor-related protein (LRP1) and has been argued to be defective in AD; however, this is still not clear [66]. Indeed, work suggesting that LRP1 can uptake Aβ directly or indirectly via ApoE or α2M binding [67–69], remains highly controversial. Additional roles of LRP1 in APP metabolism have been established more firmly. For instance, LRP1 has been shown to compete with APP for cleavage by γ-secretase [70, 71]. LRP1 also interacts with neuronal APP via the C-terminal domain and mediates endocytosis of APP, thereby increasing the production of Aβ in neuronal cells [72–74]. Another argument in support of the amyloid hypothesis is the presence of *APP* on chromosome 21, which is thought to account for the higher incidence of AD in people with Down’s syndrome [75].

However, an argument against the amyloid hypothesis in AD is the weak correlation between plaque accumulation and severity of disease progression. In fact, there is a higher correlation between the extent of tau pathology and AD, although the ratio between Aβ_42_/Aβ_40_ is a useful CSF biomarker [76–78]. In AD brain, there is an average of seven-fold more tau proteins compared to that in the normal brain and they are in an hyperphosphorylated state [79]. Tau phosphorylation is prominent at positions Ser202, Thr231, Ser235, etc. [80, 81], attributed to glycogen synthase kinase 3β (GSK3β), cyclin-dependent kinase 5 (Cdk5), JNK and MARK (microtubule-associated regulatory kinase) amongst others [82–88]. These tau kinases are believed to be activated by Aβ, suggesting AD as a secondary tauopathy [89–91].

All these observations led to the revision of the amyloid hypothesis, stating that the soluble oligomeric forms are responsible for neurotoxicity in AD and that the plaques might be in fact, protective [92]. These Aβ oligomers can form pores in the plasma membrane, therefore breaking the ionic polarity of the cell, leading to cell death [92]. This goes in line with reports that in AD brains there are more soluble Aβ_42_ monomers and oligomers than in healthy brain [93].

Over the years, the amyloid hypothesis has become quite popular among researchers and thereafter for the pharmaceutical industry. Today, the amyloid hypothesis is the most studied among researchers and is the most frequently involved in clinical trials [94]. Numerous clinical trials have been carried out based on the improvement of Aβ clearance or inhibition of Aβ production. However, most trials targeting either β- or γ-secretase have failed. Indeed, these therapies appeared quite toxic, highlighting the importance of these secretases in regulating other pathways (ex. Notch, LRP1, ApoER2, RAGE) [95–98]. More recently, many clinical trials have been based on the use of monoclonal antibodies to clear the plaques from the brain. Some have failed, while others were able to clear Aβ plaques. However, unanticipatedly, clearing the plaques was not able to reverse the cognitive decline in these patients, nor did it improve the patients’ outcomes. An explanation is that once damages induced by Aβ plaques/oligomers have already occurred, they are irreversible. Therefore, treatment should be done before the appearance of more advanced AD phenotypes.

This has led to clinical trials that enrol exclusively patients in the early stages of the disease. One of such more recent clinical trials was conducted on Aducanumab which was later fast-tracked approved by the FDA in June 2021 for use in AD patients. This drug did decrease AD biomarkers in patients; however, there was no amelioration of symptoms in the ENGAGE trial although there was a 22% decrease in cognition and function worsening in the EMERGE phase 3 trial [99]. However, the drug was later abandoned by Biogen [100, 101]. In these and previous monoclonal antibody therapies, there have been reports of amyloid-related imaging abnormalities (ARIAs) in patients [102–104]. ARIAs are a set of observations noticed during magnetic resonance imaging scan of patients taking these monoclonal antibodies. They include oedema, effusion, microhaemorrhages and superficial siderosis [104, 105]. ARIAs usually occur early in treatment, with a higher incidence in *APOE*-ε4 carriers.

More recently, clinical trials using another monoclonal anti-Aβ drug, Lecanemab developed by Eisai and Biogen or Donanemab by Eli Lilly, showed decreases of cognitive and functional decline [106, 107]. These clinical trials are the first of their kind that accomplished such definitive results and have given a new breath to the amyloid hypothesis. Lecanemab is believed to target Aβ oligomers (protofibrils), as compared to the Aβ plaques for other drugs, going in line with the revised amyloid hypothesis [92, 108, 109]. Nevertheless, there have been some cases of ARIA in patients treated with Lecanemab, with oedema and effusion (ARIA-E) occurring in 9.9% and microhaemorrhages and superficial siderosis (ARIA-H) in 10.7% of patients, and Donanemab had even more incidences [107, 108]. It can be argued that these symptoms may result from progressive weakening of the brain blood vessels because of advanced cerebral amyloid angiopathy in these patients [110–112]. However, more work is clearly needed to improve safety of these approved drugs, especially at the light of recent death reports [113].

**Fig. 2:**
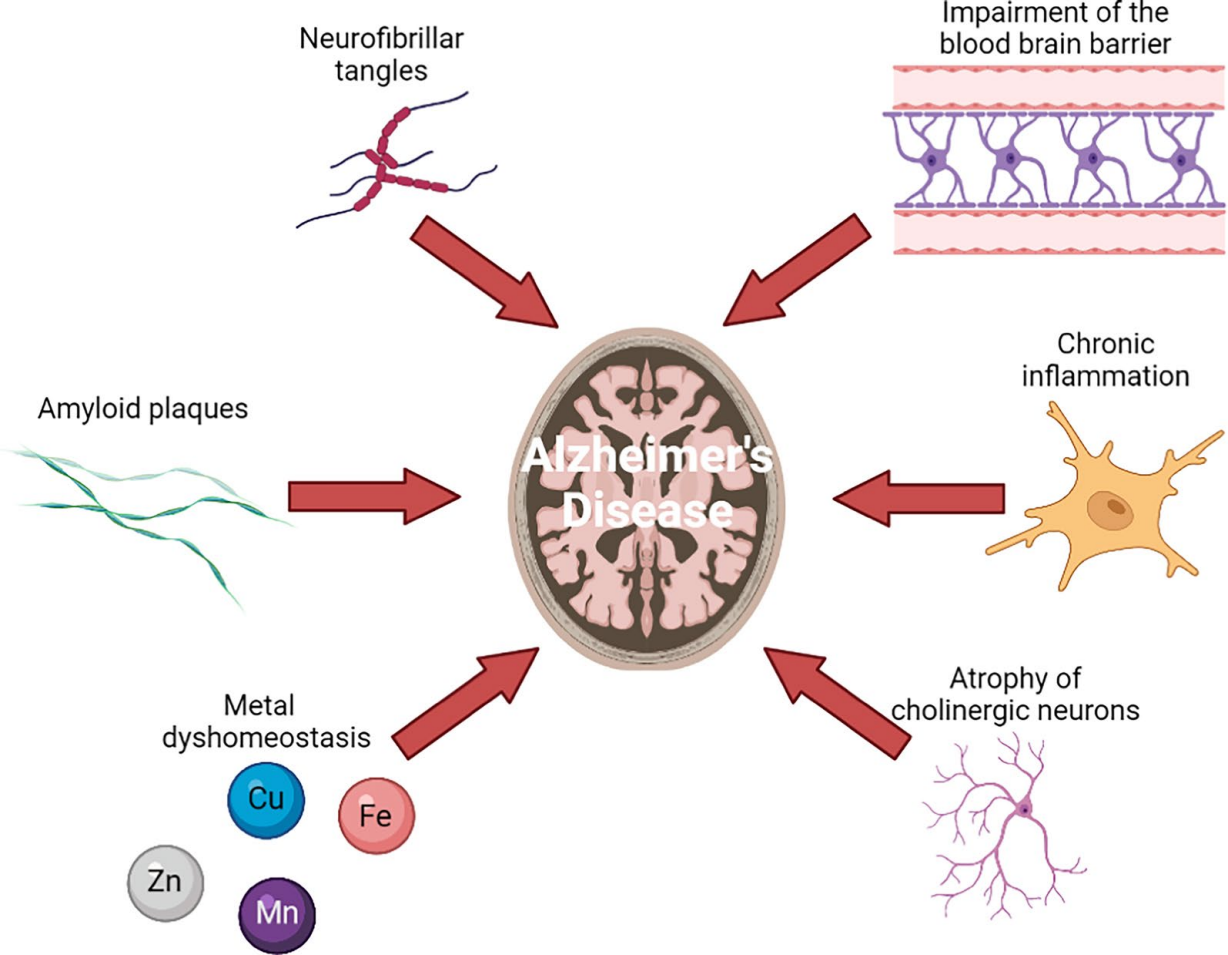
Examples of different causative agents in AD brain. Created with BioRender.com

Although these antibodies are very efficient in clearing amyloid load, linked to cognitive benefit in AD patients, their effects remain limited. This brings to light the pivotal role that other factors may play in the pathology of AD, possibly in concomitance with Aβ (Fig. 2). One such factor may be the dyshomeostasis of metal ions in AD brains, which will be developed thereafter.

## Intersections between amyloid hypothesis and metal dyshomeostasis

About a third of all enzymes are metalloenzymes, which are enzymes that need metals for their activity. Most of the metalloenzymes contain d-block metals, known for their rich chemistry. The most common of these metals in the body are Zn that serves as a Lewis acid, and Fe and Cu that serve mostly as redox active cofactors for enzymes [114, 115]. The metal dyshomeostasis theory states firstly that the natural localization of these transition metals in the brain is deregulated and secondly that pathological ligands such as Aβ can bind these metals, therefore catalysing and/or modulating their aggregation in the brain. In addition, Fe or Cu bound to Aβ is also able to catalyse the production of ROS (H_2_O_2_, O_2_^•−^ and HO^•^), which are toxic when present in excess [116, 117]. Overproduction of such ROS, known as oxidative stress, is seen from the early stages of AD, and leads to neuronal death.

Data have shown significant levels of Zn, Fe and Cu in amyloid plaques in the amygdala of AD patients. For instance, there was a four-fold increase in Cu, and a two-fold increase in Zn and Fe with a concomitant intracellular decrease of these metals [118, 119]. Miller et al. also identified Cu and Zn in amyloid plaques, with a high correlation in hotspots in AD brain [120]. However, different studies reported variable findings regarding metal levels in the brains of AD patients. A meta-analysis by Schrag et al. analysing over 30 different published articles between 1990 and 2011 showed that for bulk metal concentrations in AD brains, only Cu levels were specifically decreased in neocortex (frontal, parietal, temporal and occipital lobes), whereas Fe levels were increased in the putamen [121].

### Fe and Zn dysregulation in AD

Fe is required for essential cellular processes, such as myelination, DNA replication and generation of ATP in mitochondria, where it is a cofactor for complexes I, II, III and IV, and catalyses the transfer of electrons from NADH to O_2_ and translocates protons across the inner mitochondrial membrane in eukaryotes.

Among brain cells, Fe, transferrin (Fe-transporter) and ferritin (Fe-storage) are found predominantly in oligodendrocytes [122, 123]. In AD, there is a switch of transferrin distribution from oligodendrocytes to microglia. Additionally, around plaques there is a diffuse extracellular localisation of transferrin [124]. On the other hand, ferritin increase is associated more specifically with neuritic plaques and not with diffuse plaques or intracellular tangles [125]. Interestingly, it has been shown that Fe can increase the alpha cleavage of APP and that this is regulated via an iron response element on APP [126, 127]. Increase in Fe levels also leads to increased APP production [128]. Furthermore, APP is believed to provide complementary ferrireductase activity and might also be involved in Fe export by stabilising Ferroportin [129, 130].

Fe could be toxic in AD by both redox-mediated ROS production and ferroptosis [131]. Ayton et al. proposed the hypothesis that Fe acts upstream in AD to alter the processing of APP, or to accelerate the formation of plaques and NFTs [132]. However, only a moderate association between Fe levels in the inferior temporal lobe and the total brain NFT burden was detected.

Zn is another essential biometal that is involved in several processes including DNA synthesis, neurotransmission, and immunity. There have been many studies trying to elucidate the role of Zn in AD. For instance, an increase in extracellular Zn levels in amyloid plaques in AD patients was reported, but this has not reached a consensus in the literature [118, 121]. Nonetheless, Zn has been shown to promote the formation of amorphous, non-fibrillar Aβ aggregates [133–135]. Zn also contributes to tau pathology independently of the phosphorylation of tau, by direct binding on tau [136, 137]. Additionally, Zn transporters are found in amyloid plaques [138]. Studies have shown changes in the levels of different Zn transporters in AD [139, 140]. In transgenic mice expressing the human Swedish mutant APP, deletion of the gene encoding zinc transporter 3 (ZnT3) which is required for zinc transport into synaptic vesicles, leads to decreases of plaque loads and insoluble Aβ, accentuating a possible role of synaptic zinc in Aβ deposition [141]. Conversely, there is no change in Zn levels in the CSF or the brains of AD patients, although meta-analysis data showed that serum Zn levels are significantly lower in AD patients than in healthy controls [121, 142, 143].

### Cu dysregulation in AD

**Fig. 3:**
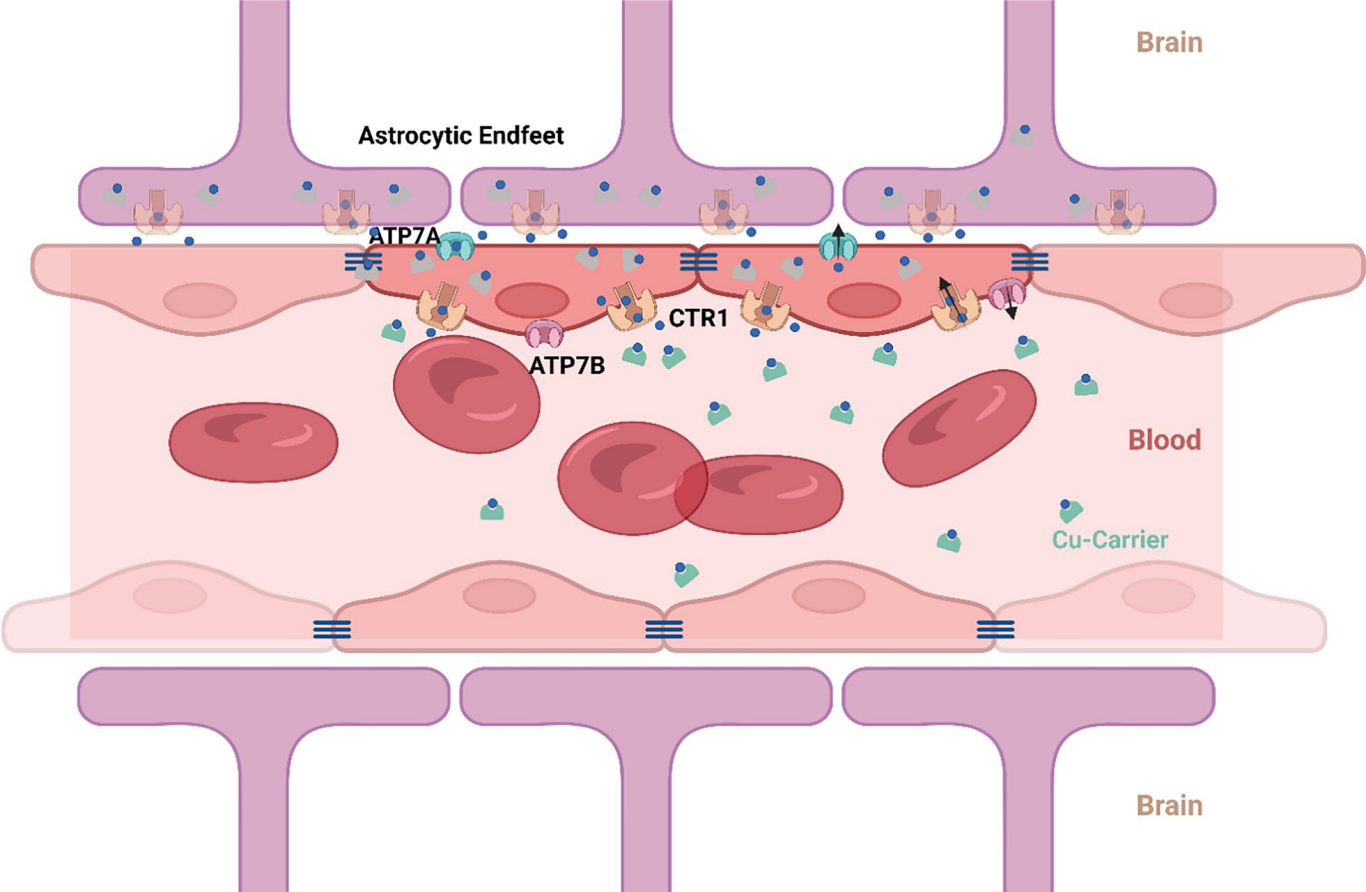
Localisation of the different Cu transporters involved in Cu importation from blood to brain. Cu is represented as a blue dot, whereas Cu carriers in the blood are green. Created with BioRender.com

Cu is an essential element for humans. It has been shown to have various important functions in the brain and is deregulated in several pathologies. Under physiological conditions, the level of Cu in the brain is primarily controlled by Cu transporters at the BBB. CTR1, present on the apical side of brain endothelial cells, imports Cu from the bloodstream. CTR1 heterozygote mice (CTR1^+/-^) have a 50% decrease in Cu content in the brain and spleen, with no changes in Cu levels in other organs, accentuating the importance of CTR1 in Cu transport into the brain [144]. On the other hand, ATP7A located on the basolateral membrane serves to import Cu into the brain parenchyma, whereas ATP7B, located on the apical membrane, serves to expel excess Cu from the brain (Fig. 3). These three transporters are also present on cells of the choroid plexus of the blood-cerebrospinal fluid barrier (BCB) [145].

ATP7A and ATP7B are related both in structure and function with 60% homology [146]. Their N-terminus possess six conservative Cu-binding domains and eight transmembrane domains that form a pore for Cu passage [146]. Cu is delivered to ATP7A/B via Atox1 [147]. The binding of Cu on ATP7A/B regulates their localization in the cell, and is believed to be phosphorylation-dependent [148, 149]. At low basal Cu levels, ATP7A/B are mainly found in the TGN, where they mainly serve to supply Cu to newly synthesized cuproenzymes, such as ceruloplasmin and dopamine β-hydroxylase [150]. ATP7B seems to be the major Cu exporter in neurons, as the level of ATP7A declines with time in the brain [151]. However, ATP7A has been shown to deliver Cu to extracellular superoxide dismutase 3 (SOD3) and to ceruloplasmin in macrophages during hypoxic conditions [152, 153]. Similar to its localization in the BBB and BCB, ATP7A is located on the basolateral membrane of enterocytes in the intestine where it pumps Cu into vesicles and serves to secrete excess Cu into the blood [154]. ATP7B is at the apical membrane where it can sequester Cu ions in vesicles of enterocytes and expels excess Cu into the lumen of the intestine for excretion [146, 155]. In the liver, ATP7B is also important for the expulsion of excess hepatic Cu, where it translocates toward the canalicular area of hepatocytes into lysosomes, thereby pumping excess Cu into the lysosome for Cu expulsion into the bile [156]. Therefore, changes in expression or function of these Cu transporters are found to have important pathophysiological repercussions.

Apart from its general role in cells, such as a catalytic center in the cytochrome *c* oxidase of respiratory chain or in the antioxidant enzyme SOD1 or SOD3, Cu is also involved in more brain-specific processes such as the synthesis of neurotransmitters (dopamine and catecholamines), myelination of neurons and synaptic function [150, 157, 158]. However, Cu is heterogeneously distributed between different brain regions with the total Cu content in human brain being approximately 5 µg/g wet brain. The brain regions containing the highest amount of Cu are the substantia nigra and locus coeruleus [159]. However, different cell types in these regions as well as other regions or different cellular compartments possess a heterogeneous distribution of Cu. For example, glial cells show higher Cu content than neurons. Equally, in neurons Cu is mainly located in the neck of the dendrite spines, where it seems to be necessary for the stability of the F-actin network [160–163]. Interestingly, unpublished data from our lab show that recombinant tau releases very slowly Cu(II), thus tau could act as a Cu sink in neurons. However, the binding of Cu(II) by tau might not be relevant intracellularly in physiological conditions, where most of Cu is believed to be in its reduced state. However, in lysosomes or in neuritic plaques, tau could easily act as a Cu sink. Nonetheless, it is possible that Cu could play a role in the stability of both actin and tau in the dendrites of neurons. These observations shine a light on the well-controlled levels and distribution of Cu in the brain.

### Cu and Aβ as partners in crime

There are many studies elucidating the interaction between Cu and Aβ or APP in the context of AD. The most convincing link comes from the presence of two Cu-binding sites in the extracellular E2 domain of APP, that have been shown to relocalize APP and may permit increased APP metabolism [164, 165]. Secondly, in APP knockout mice, there is a 40% increase in Cu levels, suggesting a possible impact of APP or its metabolites on Cu import/excretion [166]. Importantly, Cu binding to Aβ in vitro catalyses the production of ROS, more efficiently than Fe [117, 167], which may promote neuronal death. Similarly, some in vitro data show that the binding of Cu(II) to Aβ might stabilize oligomers [133, 135].

Another important link between Cu and AD-linked APP metabolites is that there is a decrease in Cu levels in AD brain. Results from several groups and meta-analysis have, however, shown a general decrease in brain Cu levels in AD using various quantitative approaches [121, 168, 169]. In the same line, recent studies have shown a decrease in Cu levels in the cytoplasm of brain cells, which is associated with a 25% increase of exchangeable Cu in the brain (probably extracellular) [170, 171]. Along with the decrease of brain Cu levels, blood Cu and non-ceruloplasmin-bound Cu (exchangeable pool) levels appear increased [121, 142, 172, 173]. Accordingly, there is a 3–4-fold increase in the risk of AD associated with the presence of excess Cu (exchangeable) in the blood [173]. A study on rabbits showed that trace amounts of Cu in the drinking water of cholesterol-fed rabbits leads to an increase in Aβ-positive neurons and plaques, as well as cognitive decline [174]. It has thus been proposed to group AD patients according to the levels of Cu in the exchangeable pool, with normal-Cu AD having exchangeable Cu ≤ 1.6 µmol/L and CuAD > 1.6 µmol/L (normal range maximum is at 1.6 µmol/L) [173]. Importantly, although there is no apparent difference between the two groups in terms of disease progression and severity, CuAD patients performed worse on neuropsychological tests such as MMSE than normal-Cu AD [173]. Interestingly, variants of *ATP7B* have been linked to CuAD [173], with the ATP7B^K832R^ (rs1061472) confirmed to have almost no Cu exporting activity [175, 176]. This is similar to Wilson disease where there is a loss of function of ceruloplasmin and ATP7B. In *ATP7B* variants, there might be an increase in exchangeable Cu pool due to the lack of excess Cu export to the bile from the liver as seen in Wilson disease [156, 177–179]. Although there is no change in Cu CSF levels in AD, there is a lower percentage of holo-ceruloplasmin (active form) in the CSF but no change in ceruloplasmin level or activity, similarly to what is seen in patient with Down syndrome [172, 180–182]. However, the lower percentage of holo-ceruloplasmin in the CSF is puzzling since this is not the case in the blood, although CSF ceruloplasmin is produced by the cells of the choroid plexus [183]. In conclusion, a shift from intracellular to extracellular brain pools of Cu might be an important aspect of the disease that has been overlooked so far.

## Cu and Aβ: in vitro chemical studies

**Fig. 4:**
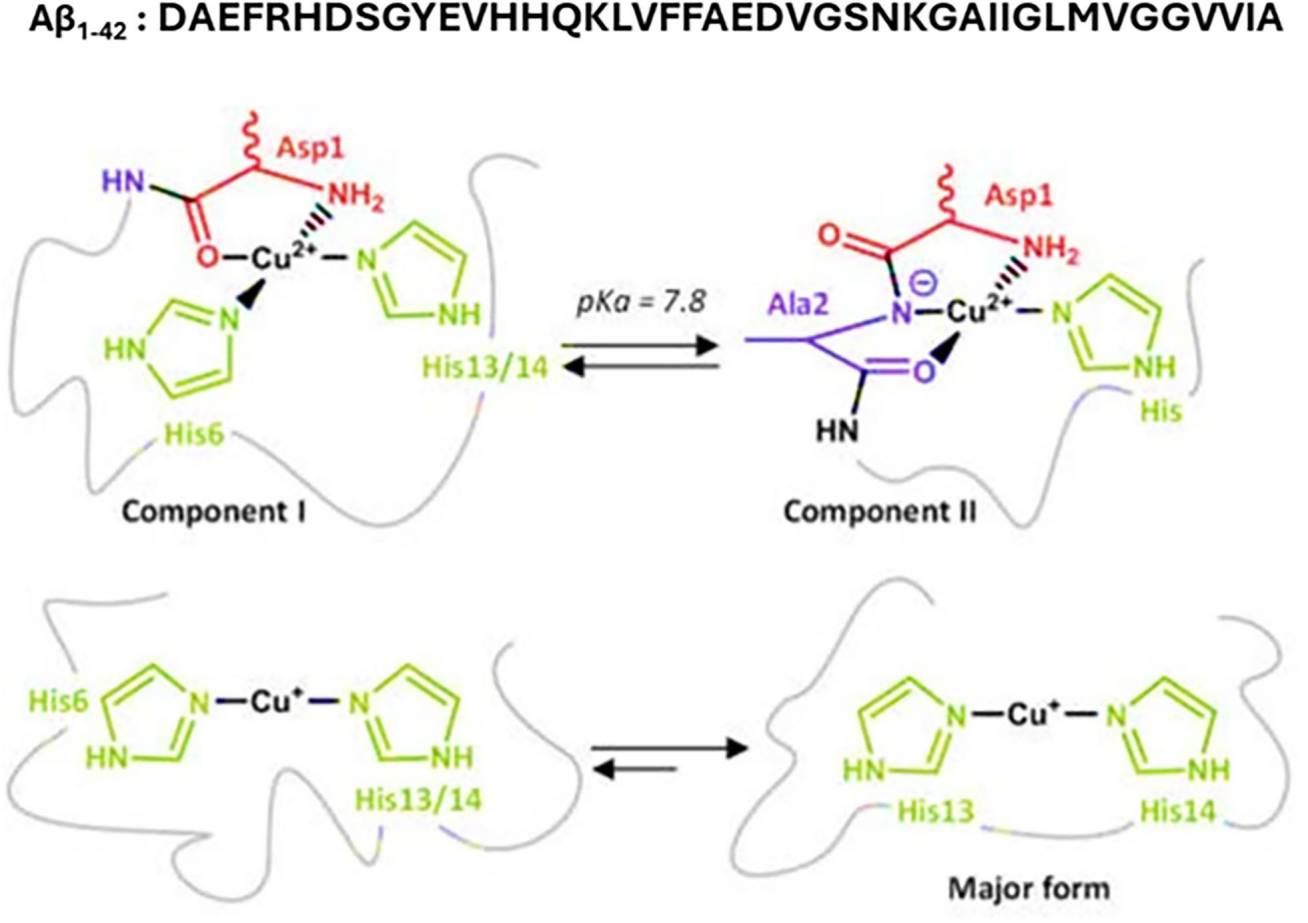
Coordination spheres of Aβ in the presence of Cu(II) (above) and Cu(I) (below). Cu(II) coordination mode consists of components I and II, while Cu(I) has a preference for the His13 and His14 linear geometry. Adapted from Cheignon et al. 2017 [117]

As highlighted in this review, Cu seems to be implicated in the toxicity observed in AD. A significant part of this is believed to come from the accumulation of Cu in amyloid plaques and from the ability of Cu(I) and Cu(II) to interact with Aβ at relatively high affinities (Log K_7.4_ ≈ 10^9–10^) and with similar or slightly higher affinity in the aggregated state [184–188]. Cu(I)-Aβ is bound mainly in a digonal coordination predominantly to His13 and His14, whereas Cu(II)-Aβ exists in a square planar geometry in an equilibrium between two components at pH 7.4 (Fig. 4). Component I is coordinated by N-terminal amine, the carboxyl of the first amide bond, the imidazole of His6 and an imidazole of His13/14 [189]. Component II is coordinated by the N-terminal amine, the first amide, a carboxyl from the Ala2-Glu3 amide bond and an imidazole [117, 189, 190].

**Fig. 5:**
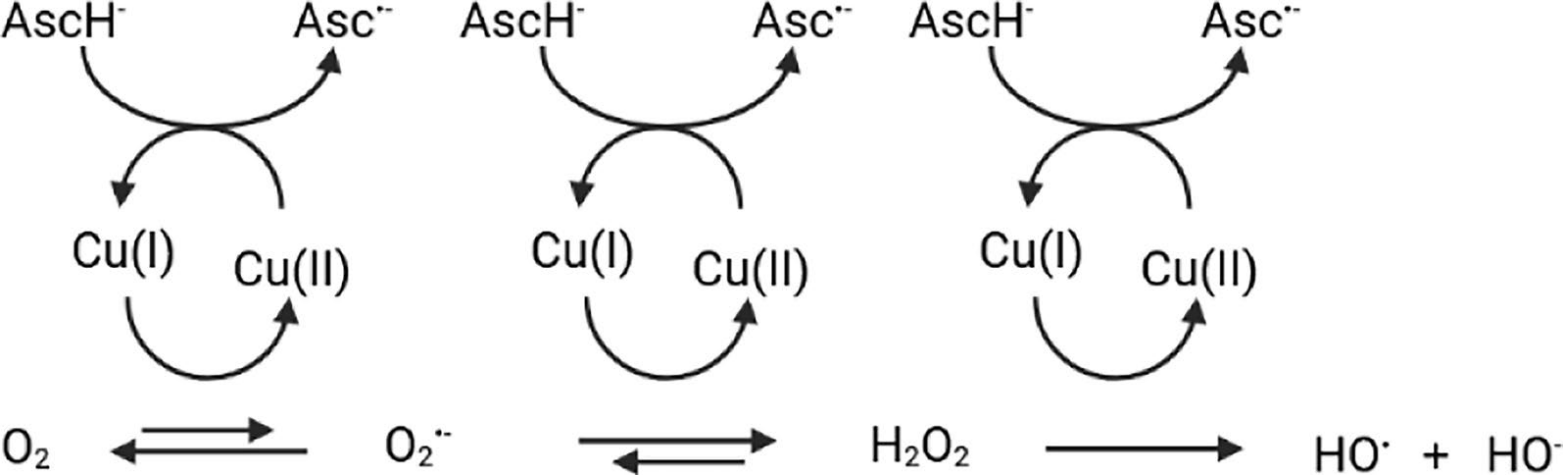
Cycling of Cu(I)/Cu(II) by ascorbate (AscH^−^) to catalyse the production of ROS

Under aerobic conditions and in the presence of reductants such as ascorbate (AscH^−^), or even dopamine [191], Aβ can cycle Cu between Cu(II) and Cu(I), thereby producing ROS (as in Fig. 5). Amongst the transition metals available in the body, Cu generates ROS most effectively since both redox states bind Aβ. However, given that the two redox states prefer different coordination geometries for Aβ binding, it was postulated that the redox reaction occurs in a pre-organized state, where Aβ can bind both redox states to permit electron transfer during the reduction or oxidation of Cu on Aβ. This pre-organized state was termed the “in-between state” and is different from the main states shown in Fig. 4. This “in-between” state is believed to represent 0.1% of the main states and has a fast exchange with the main states. Recently, a new approach including partial thermal relaxation was reported with the aim to trap such a state and characterize it by X-ray absorption spectroscopy [117, 192]. Before that, approaches to characterizing this in-between state consisted of theoretical calculation or indirect methods.

Based on the idea that the highest level of oxidation damage to Aβ is expected to be on the ligands coordinating Cu(I)/Cu(II) in the in-between state (involved in ROS production), the in-between state was predicted to coordinate Cu(I)/Cu(II) with the amine and carboxylate of Asp1, and with the imidazole of His13/14 [193, 194]. Phe19/20 and Met35 are also highly oxidized during ROS production, but another amino acid that has been reported to be oxidized is Tyr10, which is responsible for the formation of the degradation-resistant tyrosine bridges in Aβ and thus favours the formation of oligomers [195]. Cu(II) binding to two Aβ peptides could also facilitate the formation of aggregates and/or β-sheet fibril [188]. Interestingly, Cu(II) has been shown to promote the formation of Aβ_42_ protofibrils in both wild-type and fAD as well as the dissolution of mature fibres into protofibrils/oligomers, thus accentuating membrane toxicity [196, 197]. It was suggested that substochiometric Cu(II) in mixture with Aβ provides the most oligomer formation [198]. However, the bridging role of Cu(II) between two Aβ molecules remain unclear as it was suggested to be an off-path in the aggregation process [199].

Truncated or pyroglutamate form of Aβ such as Aβ_4-40/42_, Aβ_11-40/42_, pAβ_3-40/42_ and pAβ_11-40/42_ have also been found in vivo in plaques [200, 201]. Aβ_11-40/42_ accounts for around 20% of the plaque load in the brains of AD patients and accelerates the formation of fibrils by the full Aβ_1-40/42_ in vitro [201, 202]. These N-truncated species containing an ATCUN (amino-terminal Cu/Ni binding) domain (Aβ_4-40/42_ and Aβ_11-40/42_) have an extremely rapid rate of Cu binding [203–205]. They also bind Cu(II) with a higher affinity than Aβ_1-40/42_ (Log K_7.4_ ≈ 10^14^) and a high Cu(II) specificity over other metal ions (including Cu(I)). This renders the Cu(II) bound to Aβ_4-40/42_ and Aβ_11-40/42_ quasi redox inert with very low ROS production [204]. Therefore, under normal conditions, Cu should not be present on full-length Aβ peptides, except in pathological states for example in amyloid plaques where Cu is highly concentrated [118–120]. This is supported by the finding that only Aβ_4-40/42_ and Aβ_11-40/42_ are bound to Cu(II) in the CSF of healthy, MCI or AD patients [206]. More detailed literature on the chemical interaction between Cu and Aβ has been reviewed in [187, 188, 207, 208].

All these Aβ species might play complementary roles in the toxicity seen in AD. It is therefore important to rationalise their function and understand how they affect different cells in the brains of AD patients. Towards this aim, interactions of Cu ions with these forms have been studied [207].

## Link between the amyloid pathology and Cu dyshomeostasis in cells of the central nervous system (CNS)

The brain is comprised primarily of neurons (excitatory or inhibitory) as well as glial cells including astrocytes, microglia and oligodendrocytes. Their intricate proportion and localization are the basis for the spatial arrangement and function of the brain. Pelvig and colleagues report a glial population consisting of 76%–75% oligodendrocytes, 17%–20% astrocytes and 5%–7% microglia in the human cerebral cortex [209]. These characteristics are specific to each brain structure and are minutely monitored during neural development. Deregulations during development lead to an array of neurodevelopmental diseases.

In AD pathology, there is a loss of normal brain functions that arise from the loss of neurons, as well as the “over-activation” of astrocytes and microglial cells. All these alterations reduce brain plasticity, a characteristic of neurodegenerative diseases [210–214]. Therefore, it is of importance to evaluate the role each cell type plays in the development and exacerbation of AD and if or how Aβ and Cu catalyse the progression of the disease.

### Neurons: a major victim of Aβ and Cu?

Although glial cells outnumber neurons in the human brain, neurons are regarded as the central cell population that encodes and perpetuates transmission of signals between different regions. Nonetheless, glia cells play a critical supporting role [215]. In AD, cortical regions are the first regions where amyloid deposits are detectable, before they appear in the hippocampus, where the CA1 is the first region affected [9]. In these regions, neurons are the central cells affected and are regarded as the source of the pathology [216, 217]. However, there is an initial massive neuronal loss in other regions such as the cholinergic innervation of Meynert’s cells to the cortex or cells in the locus coeruleus [218–220]. Some other regions are not as much affected by the pathology, with very little cell death detected, for instance in the cerebellum [216]. However, the structures affected can be paralleled with increased presence of neuritic plaques and tau pathology. Understanding the causes of this specific neuronal vulnerability undoubtedly represents one of the greatest challenges to better understand the development of AD.

Another hallmark of AD is the presence of granulovacuolar degenerations seen in pyramidal neurons. However, these are also seen in healthy aged brains but at a much lesser extent. In AD, granulovacuolar degeneration seems to arise in the hippocampus and spreads to other structures of the temporal lobe [221]. It was shown that although granulovacuolar degeneration is associated with and proceeds cytosolic tau aggregates, they are independent of tau [222]. Of importance, granulovacuolar degeneration correlates with NFT and Aβ stages [221]. This could signify that these granulovacuoles are formed due to the presence of intracellular aggregates (tau, Aβ, or others), therefore inducing an autophagic response that is ultimately defective in AD.

Among the d-block metals, only Cu has been shown to be significantly reduced in the brains of AD patients, with neocortical regions being the most affected [121, 223]. The interaction between Cu and Aβ, and how they aggravate AD phenotypes, are not clear and obvious. However, there seems to be a link of Cu decrease in neocortical regions with cell atrophy, as well as with neuritic plaques and tau pathology [224]. Despite the general decrease of Cu levels, there seems to be concomitantly an increase of labile Cu in AD brains [170, 171]. This labile Cu could be partially bound to Aβ and cycle between its redox states, thereby producing ROS. Some ROS produced could attack cell membranes in proximity, leading to lipid and protein oxidation, whereas some ROS (ex: H_2_O_2_) could also cross the plasma membrane and attack intracellular structures.

The decrease in intraneuronal Cu could also lead to dysfunction of Cu-dependent functions, such as defect in cytochrome *c* oxidase leading to mitochondria dysfunction as seen in AD [225]. Cu homeostasis also seems to be important for the normal functioning of V-ATPase or F-ATPase, with defect in these ATPases activity leading to reduced synaptic vesicles and lysosome acidification, potentially altering processes such as neurosecretion, autophagy or mitochondrial energy production [226, 227]. It is also possible that the intraneuronal Cu deficiency leads to a decrease in catalytically active Cu/Zn-SOD, thereby hampering the ability of these neurons to deal with ROS produced from Aβ-Cu cycling.

Long-term potentiation (LTP), important for learning and memory, is clearly impaired in AD [228]. Interestingly, Aβ oligomers have been shown to inhibit hippocampal LTP in animal models [229, 230]. These oligomers can arise from Cu binding outside or inside of cells, in the presence of lower levels of Cu chaperones/binding-proteins or peptides (GSH, cysteine or metallothioneins). In vitro experiments have shown that metallothionein and GSH outcompete Aβ in Cu-binding [231, 232]. Remarkably, it has been shown that there is a significant decrease in GSH levels and other antioxidants in the frontal cortex and hippocampus of AD patients [233]. These conditions might pave the road for Aβ to “stably” bind Cu intracellularly. This model is supported by the fact that Cu facilitates the formation of oligomers and that oligomers seem to be predominantly generated intracellularly [234]. 

In normal conditions, Aβ_40_ is proposed to be formed mostly in the TGN, while Aβ_42_ forms both in the ER and in the Golgi compartments [235, 236]. Strikingly, in AD there is a decrease in the Aβ_40_/Aβ_42_ ratio, due to an increased production of Aβ_42_ [34]. In line with this, accumulation of Aβ_42_ inside human hippocampal and cortical neurons has been reported, although the exact location of this accumulation has not been elucidated [237]. Therefore, in the advent of an intracellular Cu dyshomeostasis in AD, it seems possible that the change in Aβ_40_/Aβ_42_ ratio could be related to an increased Cu level in the Golgi apparatus or the ER. BACE1, found in the Golgi apparatus or ER, has been shown to bind Cu through the Cu chaperone for SOD, copper chaperone for superoxide dismutase (CCS) [238]. This might lead to increased BACE1 expression as seen in chronically Cu-fed 3 × Tg-AD mice [239]. There could also be higher Cu levels in these compartments, thereby leading to Cu-Aβ association and formation of stable oligomers in an oxidative environment. It was equally shown that overexpression of BACE1 causes a reduction in SOD1 activity due to the competition with CCS, which was reversed by overexpression of CCS [238]. This could in part hamper the cell ability to get rid of ROS, therefore favouring the development of the pathology. Interestingly, apart from the increased expression of APP and its metabolites induced by high Cu levels, there is also an induced phosphorylation of APP at Thr668 by GSK3β, leading to axonal transport of APP [240, 241]. These vesicles seem to colocalise with ATP7A both in the presence and in the absence of Cu [240], implying that they are compartments loaded with Cu (probably parts of ER and Golgi apparatus).

Wirths et al. proposed in 2004 that intracellular accumulation of Aβ is a leading cause of cell death in AD [242]. This might be due to the intracellular accumulation of Aβ facilitating the phosphorylation of tau, hence the disruption of mitochondrial function and cell toxicity [243]. This goes in line with the observation that neuropils threads (PHFs) and NFTs precede neuritic plaques [9]. However, it has equally be shown that neurons attempt to re-enter cell cycle before manifestation of pathological hallmarks, tau and amyloid burden [244, 245]. Altogether, this strongly suggests that intracellular Aβ oligomers are also toxic on their own.

**Fig. 6:**
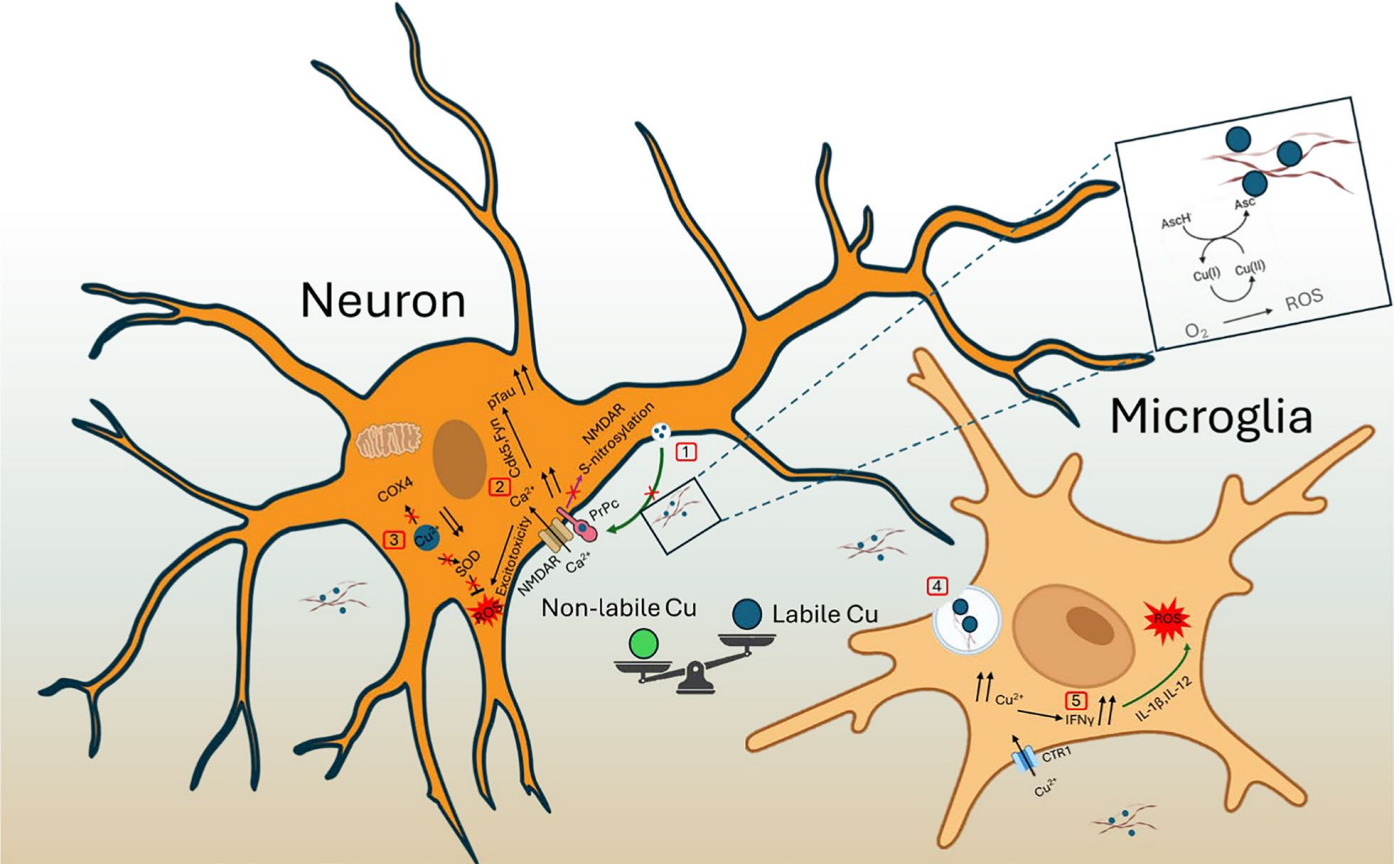
Link between Cu dyshomeostasis and AD pathology in neurons and microglia. (1) Secreted Cu from neurons is sequestered by extracellular ligands (ex: Aβ), competing with PrPc-induced *S*-nitrosylation of NMDAR, thereby promoting calcium influx. (2) Imported calcium causes increased phosphorylation of tau and increased excitotoxicity. (3) The reduced intake of Cu into neurons, induced by sequestration of Cu by Aβ, leads to less available Cu for cupro-proteins (ex: SOD, COX4), therefore lessening defence against ROS and mitochondrial dysfunction. (4) Microglia can ingest Aβ with Cu, which increases cell Cu levels. (5) This increased microglial level of Cu promotes activation of microglia through IFN-γ, exacerbating DAM profile

Nonetheless, after a certain threshold, neurons excrete the intracellularly accumulated Aβ, which can thus bind extracellular labile Cu. Regardless of their Cu-binding state, these oligomers have been shown to bind the cellular prion protein PrPc [246–248]. This binding alters the functions of PrPc, one of which is the Cu-dependent degradation of NMDA receptor (NMDAR) via *S*-nitrosylation [249]. Post-synaptic NMDAR activation leads to the ATP7A-dependent export of Cu into the synaptic cleft, which decreases NMDAR activity and protects cells from excitotoxicity [250]. Aβ oligomers can also directly provoke NMDAR-induced toxicity, by binding to NMDAR or binding to Cu. In the absence of Cu-induced NMDAR inhibition, there is an increase in intraneuronal calcium levels (excitotoxicity). These factors have been shown to lead to phosphorylation of tau through Fyn or Cdk5 (Fig. 6) [251, 252]. Equally, an increased level of intraneuronal calcium can lead to nitric oxide (^•^NO) production by nitric oxide synthase (NOS), resulting in peroxynitrite (ONOO^−^) in the presence of superoxide anion (O_2_^•−^), that can attack lipids and proteins [253]. In AD, there is indeed an aberrant expression of iNOS, eNOS and nNOS [254]. This aberrant expression may be due to brain hypofusion (i.e., reduced amount of blood flow) [255]. The hypofusion seen in AD could arise from structural abnormalities of the cerebral vasculature, for example, vasoconstriction in most areas and aberrant vasodilation (diverting blood from where it is needed), thereby restricting nutrients in parts of the brain [256].

All the possibilities discussed above may lead to aberrant functioning of neuronal cells and could on their own lead to cell death or at least alter LTP as seen in AD. This would reduce connection between synapses that can also indirectly lead to neuronal death.

### Microglia: the main effector of neurotoxicity?

White blood (leukocytes) cells such as neutrophils, monocytes, eosinophils, basophils and lymphocytes, play a vital role in the defence of the body from non-self-antigens as well as taking care of cell debris or mutated cells [257]. Given that the brain is well protected from the circulatory system by the BBB, a semi-permeable membrane, colonization of the brain by peripheral white blood cells is limited under normal conditions. The brain possesses a small array of immune cells that are quite similar to monocytes (precursor of macrophages), with different localizations such as meninges, choroid plexus, and perivascular spaces, where they play a barrier function [258].

The major type of brain immune cells are microglial cells, which account for 5%–7% of glial cells, and are found in the parenchyma of the brain where they serve as sentinels. They are derived from the yolk sac and invade the CNS prior to vasculogenesis [259]. During development they are involved in synaptogenesis and dendritic pruning of immature synapses as well as phagocytosis of cell debris [260, 261]. Under normal conditions in the adult brain, these cells patrol the brain within a few hours with extremely motile processes and protrusions surveying normal brain function [262]. However, during microglial cell activation, the cells change phenotypes to a more pro-inflammatory signature, termed ‘M1’ as for macrophages. The M1 signature is activated by interferon γ, leading to expression of pro-inflammatory cytokines such as TNF‐α, interleukin (IL)‐1β, and IL‐12 (Fig. 6) and production of ^•^NO and ROS, as well as increased expression of lysosomal proteins involved in degradation [263]. In the advent of clearance of the pathogen, microglial cells switch to the M2 signature that involves the production of anti-inflammatory signals IL-4, IL-10, IL-13 and TGF‐β. This is followed up by an upregulation of tissue repair mechanisms such as synthesis of collagen, arginase‐1, Ym1/2 and polyamines [264, 265]. However, more recently, there have been observations of different microglial signatures arising from different stimuli of microglial activation, questioning the existence of the M1/M2 activation signatures [266]. This has brought up the notion of a subset of microglia termed disease-associated microglia (DAM) as a general immunological signature for neurodegenerative diseases [267, 268].

In AD, the role of microglia is still under question. Some research proposed that their role is disease-stage dependent, being neuroprotective in early stages and neurotoxic in the later stages [269, 270]. However, DAM microglia are found surrounding amyloid plaques in AD brains and TgCRND8 mice, and a higher level of ATP7A has been found in microglia but not in neurons or astrocytes [271]. In BV-2 cells, IFN-γ was shown to be responsible for this, leading to the relocalisation of ATP7A from the TGN to vesicular structures and to an increase in *CTR1* mRNA levels, leading to Cu accumulation [271]. IFN-γ activation is Cu-dependent in this case since Cu chelation reduces ATP7A relocalisation [271]. It has equally been shown that Aβ and IFN-γ together promote the activation of microglia, associated with the production of ^•^NO and TNF-α [272]. This was confirmed in the plasma of AD patients, suggesting that IFN-γ precedes ^•^NO and TNF-α production [273].

Interestingly, subtoxic levels of Cu have also been shown to induce microglial activation and the release of ^•^NO and TNF-α via an NF-κb-dependent pathway in murine microglial BV-2 cell line. However, N-acetyl-cysteine (ROS scavenger) was able to prevent this activation [274]. In AD, there is an increase of labile extracellular Cu in the brain and studies have shown that Cu chelation by tetrathiomolybdate decreases expression of IL-1α, IL-6, IL-8 and NF-κB [275, 276]. The increase in labile Cu in AD brain might come from neurons that export Cu through ATP7A, possibly during the activation of NMDAR [250, 277, 278]. Bagheri et al. proposed that the low level of Cu seen in neurons in AD could be also due to excessive uptake by microglia during their activation [278]. Another idea could be that the lower levels of antioxidants in AD hamper the uptake of Cu from the bloodstream, explaining the generalized lower levels of Cu in the brain. This is supported by the fact that lower levels of GSH prevent Cu internalisation [279].

TREM2, a transmembrane surface protein found on microglia, is also important in the initial seeding phase of amyloid plaques, with studies showing its ability to bind Aβ oligomers (possibly stabilised by Cu). The absence or loss of function of TREM2 causes a decrease in Aβ phagocytosis and degradation [49, 50, 55]. TREM2 may thus help compact amyloid plaques, thereby reducing their perimeter and toxicity [280]. Accordingly, in the absence of TREM2, plaques are more diffuse with a loose plaque structure, associated with the absence of increased IL-6 and MIP-1α levels [49, 55, 56].

Lastly, Cu activates microglial inflammasome, leading to secretion of pro-inflammatory factors (IL-1β, TNF-α) and apoptosis-associated speck (ASC), which are suspected to play an important role in the early stages of AD [281, 282]. ASC favours seeding of Aβ, as ASC specks are found in the core of plaques and accelerate oligomer formation and possibly the spreading of amyloid pathology [283].

### Astrocytes: a secondary effector for neurotoxicity?

Astrocytes are star-shaped glial cells present in the brain and originate from neuroepithelium-derived radial glia, which are universal neural progenitor cells, by direct transformation or by the generation of intermediate progenitors [284–286]. Astrocytes are very important in the spatial temporal functioning of the brain as they control ionic homeostasis, synthesis and clearance of neurotransmitters, plasticity, formation and maturation of synapses as well as elimination of unnecessary synapses [287–291]. They form a network between themselves via gap junctions and in response to synaptic activity, they can release gliotransmitters which can act on neurons (synaptic plasticity) and vascular smooth muscle tone (blood flow) [292].

Astrocytes exist in both grey and white matter, known as protoplasmic and fibrous astrocytes, respectively. The latter send out long processes that contact axons at the node de Ranvier and contact the blood vessels, hence forming the end feet [293]. On the other hand, protoplasmic astrocytes make contacts between pre- and post-synaptic neurons, forming the tripartite synapse, and also make contacts with brain capillaries. Unlike fibrous astrocytes, protoplasmic astrocytes arrange themselves in domains, with a certain degree of minimized overlap, giving rise to various morphological shapes of astrocytes [294]. It is of note that human astrocytes are quite different from rodent’s, therefore animal models will not encompass all the characteristics of human astrocytes in disease models. Some of these features include the facts that human protoplasmic astrocytes are 2.6 times larger in diameter and propagate calcium waves fourfold faster than those from mice [295]. Additionally, in primates, there are two additional types of astrocytes, interlaminar and varicose-projection astrocytes, in the cortex [295]. However, in both AD mouse models and human patients, there is an atrophy of astroglia (both of cell volume and of processes), accompanied by reactive astrocytes characterised by increased volume and an increase in glial fibrillary acidic protein (GFAP) expression [296, 297].

GFAP is a type III intermediate filament protein that is a known marker for astrocytes. The expression of GFAP has been shown to increase in pathological conditions including at the late stage of AD in transgenic mouse models as well as in AD postmortem tissues. However, GFAP also increases with age, and it has been shown to be sensitive to the presence of ROS in the company or absence of microglial cells [298]. This sheds light on the existence of ROS production in the parenchyma of healthy individuals. However, under pathological conditions, ROS production is far superior. Interestingly, in 3 × Tg-AD mice, a reduction of GFAP expression at initial stages of AD has been shown, followed by an increase at later stages. Nonetheless, astrocytes surrounding plaques are clearly hypertrophic (reactive) with an increase in surface and volume [296]. These reactive astrocytes seem to lose their function, such as in new synapse formation and synaptic pruning [299]. They can be activated by a combination of IL-1α, TNFα, and C1q but not IL-1β, released by microglial activation [299]. Intriguingly, IL-1α induces production of APP and α-secretase-mediated APP metabolism in human astrocytic cells [300]. This might be neuroprotective, since the α-secretase-mediated APP metabolism is believed to have a protective effect in AD.

The role astrocytes play in AD appears complex and it is not clear if they play a principal or a secondary role (reacting to microglial activation). A factor nailing the importance of astrocytes in AD pathogenesis is that several AD risk factors are genes mostly expressed in astrocytic cells. These include *APOE* and Clusterin (*APOJ*), with the latter being important in the importation of lipids/Aβ in the brain [301]. LRP1 is another protein affected, which is highly expressed in glial cells and astrocytes, and helps in the clearance of Aβ [302, 303]. Interestingly, in aging mice chronically dosed via their drinking water with Cu (2 µmol/L), Cu accumulated in the brain capillaries but not in the parenchyma and that this accumulation led to a decrease in LRP1, possibly through PrPc-induced *S*-nitrosylation [304]. Conversely, in APP^sw/0^ mice, there was an increased Cu levels in both the parenchyma and capillaries which might be explained by a compromised BBB. Nonetheless, in these mice there was a lower LRP1 level, accompanied by higher Aβ load. This might be relevant in AD, where there is a higher level of non-ceruloplasmin Cu, which might affect the ability of astrocytes to export Aβ through LRP1. Additionally, Aβ oligomers can activate astrocytes in mice possibly through binding directly to C1q (released by microglia), leading to activation of NF-κB and release of C3 complement (increased in AD patients), which are toxic to both microglia and neurons [299, 305, 306].

Some of the most important astrocytic functions for neurons are the uptake of glucose and release of lactate, the uptake of glutamate and release of glutamine and the uptake of GSH precursors and release of GSH, all of which are deregulated in AD [307]. In cultured astrocytes there is an increased release of GSH and an increased glucose consumption in the presence of Cu [308]. Additionally, Cu(II)Aβ, as well as Zn(II)Aβ, have been shown to decrease glutamate uptake and increase lactate release (in the absence of mitochondrial dysfunction) by astrocytes [309]. The increase of GSH secretion could be explained as a response to suppress ROS production, whereas the increase in glucose metabolism is more complex to understand. However, it has been shown that Cu stabilises HIF-1α through a CCS-dependent mechanism and leads to the expression of lactate dyshydrogenase for conversion of pyruvate to lactate under normoxic conditions, apart from VEGF upregulation for angiogenesis [310, 311]. It can thus be argued that in AD, due to the reduced levels of GSH in regions affected [233], there is a lesser import of Cu in astrocytic cells from the blood as well as a lack of Cu-dependent stabilization of HIF-1α, therefore leading to decreased levels of lactate dehydrogenase, and thus reduced lactate production. Paradoxically, increased levels of ROS, which can be generated by Cu, should also increase activation of nuclear factor erythroid 2-related factor 2 (NRF2) that increases the expression of antioxidant genes including GSH [312].

## Therapeutic molecules and mechanisms of action

### Clinical trials: metal dyshomeostasis regulators

**Fig. 7:**
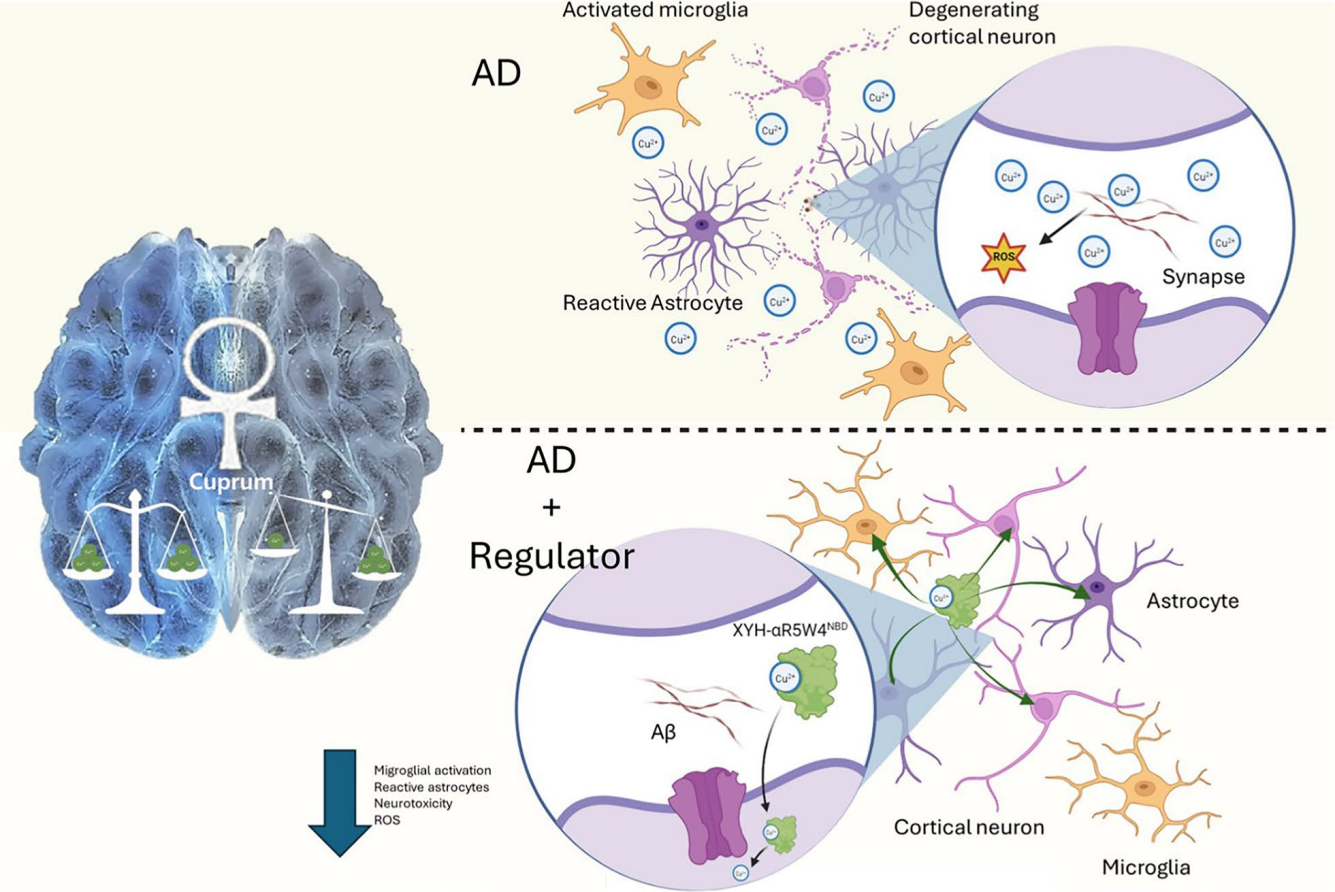
Scheme illustrating the mechanism of action for a Cu dyshomeostasis regulator. In AD brain, there is an excess amount of exchangeable Cu pool which leads to the generation of ROS. Potentially, treatment with a Cu dyshomeostasis regulator relocalises this excess Cu back into cells in the brain

In the healthy brain, there is a heterogeneous distribution of metal ions, which is tightly regulated in a localisation-dependent manner. Given the dyshomeostasis of metal ions in AD, clinical trials attempting to increase or decrease metal ion levels have been tested (Fig. 7).

Zinthionein is a formulation containing Zn and cysteine that demonstrated no significant benefit on any of the cognitive tests in an AD context [313]. Cu supplement was also tested in patients with mild AD; however, there was no benefit on AD evolution [314]. D-Penicillamine, a reducing agent and chelator used in Wilson disease to chelate excess Cu, was used in a pilot phase trial. The chelate did decrease ROS indicators in the blood but did not have a beneficial effect on cognitive decline in AD [315]. This is however not surprising since the chelate does not correct the neurological symptoms due to excess Cu in Wilson disease [316].

A few clinical trials based on disrupting the interaction between protein and Fe, Cu or Zn have also been enacted. The first one showed that the Fe(III) chelator desferrioxamine slows AD progression in regard to cognitive decline. However, no follow up was carried out and there were some issues with the trial, including the authors initially aiming for aluminium and the control not being double-blinded [317]. Two additional clinical trials using clioquinol and PBT2 have been conducted with the aim of redistributing Cu/Zn in the brain [318–320]. Clioquinol is a Cu and Zn chelator that can cross the BBB. The trial was intended to slow the progression of amyloid plaques by removing Cu and Zn, thus reducing Aβ aggregation and their toxic effects. Although the clinical trial showed a decrease in plasma Aβ, the trial was stopped due to a toxic impurity associated with the compound [319]. The clioquinol derivative, PBT2, was later developed and was shown to reduce the level of Aβ in CSF but without significant improvement of cognitive parameters. It also had no effect on plasma biomarkers of AD or serum Zn and Cu concentrations [318, 320]. Criticisms of these studies are that the molecules used have other functions apart from the chelation of Cu and Zn, and the lack of specificity for either metal ion as well as potential binding to Fe, which obviously make it difficult to distinguish the role of Cu, Zn or Fe in AD. It is also possible that these molecules induce an excess import of Cu into neurons, leading to cuproptosis as seen for the elesclomol derivatives [321].

### Proposed general mechanisms of action for Cu dyshomeostasis regulators in AD

Extensive studies have been carried out on the metal-protein attenuating properties of 8-hydroxyquinolines (e.g., clioquinol and PBT2) and Bis(thiosemicarbazonato) such as glyoxal-bis N4-methylthiosemicarbazonato (GTSM). These molecules proved to be capable of importing Cu into cells, leading to the decrease in extracellular Aβ in both cellular and animal models of AD [241, 322]. However, of the listed compounds, only GTSM might be selective for Cu(II).

The clinically tested clioquinol, as well as GTSM, in complex with Cu, can activate the PI3K/Akt pathway that leads to the phosphorylation of GSK3β at Ser9 [241, 323]. GSK3β is constitutively active in cells and is abundant in the CNS, known to inhibit glycogen synthase via phosphorylation [324, 325]. GSK3β is inactivated by insulin/IGF-1. The latter activates PDK1 (3-phosphoinositide-dependent protein kinase-1) which then activates protein kinase B that in turn inactivates GSK3β. However, PKC and MAPK are also known to inhibit GSK3β [326–328].

Interestingly, activated GSK3β has been shown to increase Aβ production by interacting with APP, BACE1 or PS1 [329]. GSK3β has also been shown to be an upstream activator of Fyn leading to degradation of NRF2 [330]. Therefore, the GSK3β-dependent activation of Fyn might equally phosphorylate tau, as there is an increase in the presence of Fyn in neurons containing phosphorylated tau [331, 332]. Fyn kinase is also known to phosphorylate NR2A/B subunits of NMDARs, thereby stabilising NMDAR at the synapse, possibly leading to excitotoxicity [333]. Fyn can also be activated by Aβ oligomers through a PrPc-specific signalling [247].

At another level, the redistribution of Cu alone is another mechanism of action on its own. Cu is believed to stabilize Aβ oligomers, that can lead to the formation of pores in cells, thus increasing intracellular calcium levels, leading to cell toxicity. Equally, Aβ oligomers are known to lead to “over-activation” of NMDARs either by directly binding Cu or by instigating an “open” conformational switch, thereby increasing intraneuronal calcium levels. High levels of calcium are thought to activate Fyn or Cdk5, believed to phosphorylate tau [251, 252]. Additionally, increase in labile Cu is also thought to be able to degrade LRP1 through PrPc-dependent *S*-nitrosylation [304]. This might be the cause of down-regulation of LRP1 in the capillaries of AD patients leading to accumulation of Aβ in the brain. Additionally, Cu increases Aβ production, reduces Aβ clearance and degradation in the brain parenchyma [304, 334]. This shines light on the Aβ-Cu axis and brings up the notion of where in the brain Cu is toxic. We believe that this toxicity comes from both the decreased activity of Cu proteins and an increase in the extracellular labile Cu pool, therefore leading to ROS production. Therefore, Cu dyshomeostasis regulators may correct this toxicity by clearing labile Cu pool and restore normal Cu homeostasis.

## Conclusion

Correct regulation of Cu levels in the CNS and in the body as a whole is vital. The dyshomeostasis of Cu as uncovered in AD likely plays a crucial role in the dysfunction of different brain cell populations. Neuronal hyperactivity precedes neuronal loss in AD [335]. This hyperactivation of neurons is thought to be in part due to sustained NMDAR activity leading to Ca^2+^ imbalance [336]. It has been shown that Cu dyshomeostasis [248, 249], Aβ and tau can all drive this phenomenon, as reviewed here [336]. Therefore, clearing excess extracellular Cu and redistributing it into neuronal cells may regulate NMDAR hyperactivity. This will also increase the resistance of these neurons to ROS, due to the role of Cu in oxidative defence. Another level where the redistribution of Cu from the outside to the inside of cells is important, is the microglia-activating property of extracellular Cu [274]. Cu is needed for IFN-activation and that IFN and Aβ together promote the microglial activation [271–273]. As well, hyperactive cells give an “eat me” signal to microglial cells, which can lead to sustained activation as seen in AD [272, 335, 336]. Therefore, depriving Cu from microglial cells could help reduce cytokine production and thus microglial activation. The simultaneous redistribution of non-physiological extracellular Cu and importation of Cu into neuronal cells, reduce Cu-induced microglial activation and could be an interesting therapeutic venture.

New studies showing the effect of ionophores with selectivity towards Cu are emerging [337–344]. However, what is of note in this proposed therapy is that these new-generation Cu transporters should bind selectively and specifically to Cu^2+^, the extracellular form. They should also have a moderate affinity, as not to compete with physiological Cu chaperones or cuproproteins, but strong enough to withdraw Cu from promiscuous Cu ligands (e.g. Aβ), and importantly, with no secondary activities. Such potential Cu transporters include, for instance, peptide-based Cu shuttles capable of recovering Cu from Aβ aggregates, preventing cell toxicity and increasing bioavailable intracellular Cu levels [328, 331], or molecules capable of retrieving Cu from Aβ but not endogenous ligand with similar affinity (ex: PrPc) [345]. Another important detail to take into account in the design of these Cu transporters is that excessive increase of Cu in neurons can lead to cuprotoxicity as seen for molecules such as elesclomol, GTSM, clioquinol, likely resulting from rapid increase in mitochondrial Cu levels [321]. Interestingly, different peptide-based Cu shuttles display different kinetics of Cu release, which may be of particular interest regarding this aspect [331]. Therefore, increase of Cu levels in compartment where it is useful (ex: Golgi, for incorporation in cuproproteins) and with an ideal kinetic, should be desired.

Understanding how Cu dyshomeostasis is present in individual cell types or cell compartments is thus relevant, so as to be able to target specific cell types/compartments in re-equilibrating Cu levels in AD. Therefore, new studies will need to be carried out to answer this question, and new molecules capable of correcting Cu dyshomeostasis are needed. Another key aspect that will be needed to be taken into consideration in future clinical trials involving Cu-shuttles and metal transporters in general is to stratify AD patients for whom metal dyshomeostasis could contribute to the progression of the disease. Finally, like the most recent trial, these molecules are likely to work best before neuronal death and thus administered before the first symptoms are detected. Progress in early AD diagnosis and progress in Cu sensors are therefore also key to therapeutic progress.

## Data Availability

Not applicable.

## Abbreviations

α-syn: α-Synuclein
α2M: α-Macroglobulin
AD: Alzheimer’s disease
ALS: Amyotrophic lateral sclerosis
APP: Amyloid precursor protein
AscH^−^: Ascorbate
ATOX1: Antioxidant 1 Cu chaperone
Aβ: Amyloid-β
BBB: Blood–brain barrier
BCB: Blood-cerebrospinal fluid barrier
CAA: Cerebral amyloid angiopathy
CCS: Copper chaperone for superoxide dismutase
COX: Cytochrome C oxidase
CSF: Cerebrospinal fluid
DβH: Dopamine β-hydroxylase
GSH: Glutathione
GTSM: Glyoxal-bis N4-methylthiosemicarbazonato
PD: Parkinson’s disease
PHFs: Paired helical filaments
PrP: Prion protein
ROS: Reactive oxygen species
SOD: Superoxide dismutase
